# Developing a Simulation to Foster Prospective Mathematics Teachers’ Diagnostic Competencies: the Effects of Scaffolding

**DOI:** 10.1007/s13138-022-00210-0

**Published:** 2022-07-27

**Authors:** Christian Schons, Andreas Obersteiner, Frank Reinhold, Frank Fischer, Kristina Reiss

**Affiliations:** 1 Heinz Nixdorf-Chair of Mathematics Education, Technical University of Munich, Marsstraße 20, 80335 Munich, Germany; 2 Institute for Mathematics Education, Freiburg University of Education, Freiburg, Germany; 3 Department of Psychology, Ludwig Maximilians University Munich, Munich, Germany

**Keywords:** Diagnostic competencies, Scaffolding, Diagnostic activities, Simulation-based learning, Teacher education, Misconceptions, Diagnosekompetenzen, Scaffolding, Diagnostische Aktivitäten, Simulationsbasiertes Lernen, Lehrerbildung, Fehlvorstellungen

## Abstract

To assess individual students’ abilities and misconceptions in mathematics, teachers need diagnostic competencies. Although research has addressed the quality of teachers’ diagnostic competencies in recent years, it is not very clear how to foster these competencies effectively in the course of prospective teachers’ university education. Research suggests that simulations with instructional support are promising tools for fostering complex competencies. We have developed a simulation that aims at measuring and fostering prospective primary school teachers’ competencies to assess students’ mathematical abilities and misconceptions based on their written task solutions. In this study, we analysed data from prospective primary school mathematics teachers who used one of three different versions of the simulation. Two versions contained a specific type of scaffolding, while the third version did not contain scaffolding. Specifically, the two scaffolding types were *content-related scaffolding* that emphasized the use of specific pedagogical content knowledge, and *strategic scaffolding* that emphasized diagnostic activities. The results suggest that integrating scaffolding into the simulation did not substantially influence participants’ overall perception of the simulation regarding presence, authenticity, or perceived cognitive load. Compared to participants in a control group without intervention, participants who used the simulation with scaffolding had higher diagnostic accuracy regarding overall assessment of students’ competence level. However, only content-related scaffolding but not strategic scaffolding or no scaffolding tended to improve participants’ competence in identifying students’ specific misconceptions. The results provide a first empirical basis for further development of the simulation.

## Abstract (de)

Um die mathematischen Fähigkeiten und Fehlvorstellungen einzelner Schülerinnen und Schüler einzuschätzen, benötigen Lehrkräfte Diagnosekompetenzen. Obwohl sich die Forschung in den letzten Jahren vermehrt mit Diagnosekompetenzen von Lehrkräften befasst hat, ist noch weitgehend unklar, wie man diese effektiv während der universitären Lehramtsausbildung fördern kann. Forschungsergebnisse legen nahe, dass Simulationen mit instruktionalen Unterstützungsmaßnahmen eine vielversprechende Möglichkeit bieten, komplexere Kompetenzen zu fördern. Im vorliegenden Projekt wurde eine Simulation entwickelt mit dem Ziel, die Fähigkeiten angehender Grundschulehrkräfte zu messen und zu fördern, mathematische Kompetenzen und Fehlvorstellungen von Schülerinnen und Schülern anhand von schriftlichen Aufgabenlösungen einzuschätzen. In dieser Studie untersuchten wir Daten angehender Grundschullehrkräfte, die mit einer von insgesamt drei verschiedenen Versionen der Simulation arbeiteten. Zwei der Versionen enthielten eine bestimmte Art von Scaffolding, wohingegen eine dritte keinerlei Scaffolding beinhaltete. Die beiden Scaffoldingarten waren inhaltsbezogenes Scaffolding, das spezifisches fachdidaktisches Wissen unterstützte, und strategisches Scaffolding, das diagnostische Aktivitäten unterstützte. Die Ergebnisse zeigen, dass Scaffolding keinen substantiellen Einfluss darauf hatte, wie die Teilnehmerinnen und Teilnehmer die Simulation hinsichtlich Presence, Authentizität oder Cognitve Load erlebten. Im Vergleich mit einer Kontrollgruppe ohne Intervention schätzten die Teilnehmer, welche die Simulation mit Scaffolding verwendeten, die mathematische Kompetenz akkurater ein. Allerdings zeigte nur das inhaltsbezogene Scaffolding, jedoch nicht das strategische Scaffolding eine Tendenz, das Erkennen von Fehlvorstellungen zu verbessern. Die Ergebnisse stellen eine erste empirische Basis für die Weiterentwicklung der Simulation dar.

## Introduction

Assessing individual students’ abilities and misconceptions in mathematics is an important facet of teachers’ diagnostic competence. Many empirical studies focused on the accuracy of teachers’ assessments. Research additionally aims at understanding the underlying diagnostic processes (Artelt and Rausch 2014; Herppich et al. 2018; Loibl et al. 2020). The accuracy of teachers’ assessments appears to depend on specific affordances of a situation and on references teachers use for their judgments (Hoge and Coladarci 1989; Südkamp et al. 2012). To date, we do not fully understand the factors that influence teachers’ diagnostic processes and outcomes, or the most effective instructional ways of fostering (prospective) teachers’ diagnostic competencies during teacher training (Leuders et al. 2022; Praetorius et al. 2012). Research suggests that digital simulations with instructional support are promising tools for fostering complex competencies that require the application of theoretical knowledge in practical situations (Heitzmann et al. 2019). Because diagnosing can be considered such a complex competency, digital simulations could effectively complement regular teacher training.

The general aim of the research presented here is the development of a digital simulation that can be used to assess and foster prospective primary school mathematics teachers’ diagnostic competencies. The simulation facilitates the assessment of virtual primary school students’ mathematical competencies based on their written solutions to mathematical tasks. To provide targeted support, we modified an existing version of the simulation (as described in Wildgans-Lang et al., 2022) and implemented scaffolding, that is, instructional support provided during the learning process. In this article, we were specifically interested in the effects of this scaffolding.

### Diagnostic Competence in Teacher Education

Teachers’ diagnostic competence includes skills for accurately assessing students’ learning processes and outcomes as well as the challenges in learning situations to initiate adequate professional actions (Artelt and Gräsel 2009; Helmke et al. 2004; Lorenz 2011; Schrader 2009). Accordingly, teachers need to assess learners’ abilities as well as the challenges they may face in learning situations, such as task difficulties (Karst 2012; Ostermann et al. 2015). Assessing these challenges is particularly relevant for mathematics teachers as they often evaluate students’ abilities by selecting mathematical tasks and evaluating students’ task solutions. In such diagnostic situations, teachers need to analyse relations between an individual student’s task solution and relevant task features that can potentially provide evidence about this student’s mathematical competency (Anders et al. 2010; Artelt and Gräsel 2009; Helmke and Schrader 1987). Moreover, teachers need to recognise students’ mistakes in their solutions and need to interpret them accurately against the background of learning goals that are more or less clearly defined (Padberg 1996; Radatz 1980).

To study diagnostic competence in this scenario, a theoretical framework is necessary that allows mapping between relevant task features and students’ written work on the one hand and students’ mathematical competencies on the other. One type of such a framework is a theoretically sound and empirically validated competence model that can provide a normative reference, and that may also support teachers in diagnostic situations (Reiss and Obersteiner, 2019). Such competence models may be helpful for assessing both, the students’ general competence level (as defined by the model) and their specific misconceptions (derived from the descriptions of individual competence levels according to the model). For these reasons, we used a mathematical competence model as a basis for the assessments in the current study.

Investigating and supporting teachers’ diagnostic competencies requires appropriate diagnostic situations (Leuders et al. 2018). The reason is that diagnostic competence does not only require theoretical knowledge, but also requires utilizing this knowledge in practical situations (Klug et al. 2013; Reinhold 2018). Because real-life contexts are often not suitable for that purpose, simulation-based learning environments seem to be more promising (Chernikova 2020b; Codreanu et al., 2021; Heitzmann et al. 2019; Wildgans-Lang et al. 2020).

### Developing Simulations for Learning Purposes

Simulations have been used successfully for training purposes in educational contexts, especially in medical education (e.g., Cook et al. 2011; Issenberg et al. 2005), and in the acquisition of complex problem-solving skills (e.g., Funke 1988). A simulation can be defined as a model of a natural system with features that can be manipulated (Heitzmann et al. 2019). Simulations in research on learning and instruction aim at providing authentic problems that allow different strategies to be applied (de Jong and van Joolingen 1998; van Merriënboer and Paas 2003), and therefore prepare learners for real challenges (Dieker et al. 2014; Grossman et al. 2009). From a general learning science perspective, several factors need to be considered when developing effective simulations.

One factor is *presence* (Sauter et al. 2013), which is a measure of the extent to which individuals immerse themselves in a situation (Schubert et al. 2001). A second, and related factor is *authenticity* (Seidel et al. 2010), which refers to the extent to which individuals perceive a situation as realistic. Both factors relate to the degree to which the simulation approximates a real situation (Codreanu et al. 2020; Dieker et al. 2014; Grossman et al. 2009). A challenge when developing simulations for learning is that there can be a trade-off between authenticity and instructional features implemented in the simulation. Because real situations do not include any such features, instructional features could reduce the degree to which the situation is perceived as authentic. A third factor that should be considered in simulations for learning purposes is the *cognitive load* induced by the simulation (Sweller 1989; van Merriënboer et al. 1992). According to cognitive-load theory, especially novice learners—whose knowledge is often not well organised—can be overburdened by the complexity of a new problem (Renkl and Atkinson 2003). For that reason, the individuals’ extraneous cognitive load that is caused by operating with the simulation itself and that is not related to the learning task should be low, so that learners have enough cognitive capacities for the actual learning tasks (Codreanu et al. 2020; Sweller 2005). De Jong and van Joolingen (1998) argue that research should consider the potential effects of instructional support on cognitive load. This seems particularly important when comparing the effects of different *kinds* of support, because they may differ in the cognitive load they impose on learners.

Few studies used simulations in teacher education to foster professional competencies, and the contexts of these studies vary widely (Chernikova 2020b). Studies that addressed teachers’ diagnostic competencies mostly used simulations as a tool for analysis rather than for instruction (Codreanu et al. 2021; Kron et al. 2021; Shaughnessy and Boerst 2018; Wildgans-Lang et al. 2020). Therefore, little is known about how to construct effective simulations for supporting diagnostic competencies in mathematics. Assessing students’ mathematical competencies and misconceptions based on selecting and evaluating tasks is a diagnostic situation that can be presented in simulations and can be regarded as sufficiently complex (Heinrichs and Kaiser 2018, Philipp 2018). The simulation used in this study presents such a task-based diagnostic situation (for details, see 2.1); it requires several activities that can be described as a diagnostic process.

### The Diagnostic Process

**Fig. 1:**
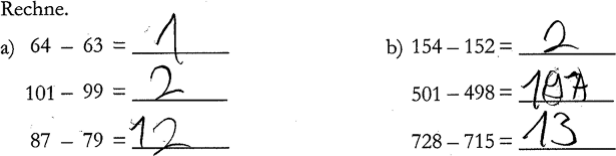
Example of an original VERA‑3 task solution showing a misconception in subtraction with decadal transition

According to Helmke et al. (2004) and Schrader (2008), assessing is an iterative process that includes repeated evaluation of evidence and reflection of initial judgements in the face of additional information. *Diagnostic activities* can be considered as an instance of scientific reasoning and argumentation, and the model by Fischer et al. (2014) describes typical activities in the process of scientific reasoning and argumentation. Heitzmann et al. (2019) specified these activities with regard to assessment situations. These diagnostic activities are: (1) problem identification, (2) questioning, (3) generating hypotheses, (4) selecting tasks, (5) evaluating solutions, and (6) drawing conclusions. We illustrate these activities based on an example of a student’s solution (see Fig. 1) to a mathematical task that was used in the large-scale study VERA‑3 (Stanat et al. 2012): First, teachers identify that the student has solved incorrectly two of six subtraction problems presented in Fig. 1 (1), whereupon they ask themselves what the reason for these errors could be (2). They state the hypothesis that the student might systematically subtract the smaller from the larger number digit by digit in multi-digit subtraction tasks (3). Teachers select another multi-digit subtraction task because they want to check their hypothesis (4) and evaluate the student’s solution to this new task (5). Finally, they draw conclusions about the student’s potential misconception (6).

There is evidence that carrying out the diagnostic activities in a strategic way (e.g., evaluating evidence based on hypotheses, as illustrated above) is important for successful learning in problem-based learning environments (Codreanu et al. 2021; de Jong and van Joolingen 1998; Schauble et al. 1991; Wildgans-Lang et al. 2020). In addition to the competency of carrying out diagnostic activities, assessing student’s competencies in a specific domain also requires professional knowledge.

### Professional Knowledge as a Prerequisite for Student Assessment

Various models describe (mathematics) teachers’ professional knowledge and mostly include content knowledge and pedagogical content knowledge (e.g., Ball et al. 2008; Shulman 1987; Weinert et al. 1990). From a theoretical point of view, teachers need to integrate their knowledge from different facets to master diagnostic situations (Brunner et al. 2011; Helmke 2017; Weinert et al. 1990). Although empirical evidence for the relationship between diagnostic competence and professional teachers’ knowledge is still lacking (von Aufschnaiter et al. 2015; Schrader 2014), there is agreement that content-related knowledge facets are necessary prerequisites for making accurate assessments.

From a mathematics educational perspective, content knowledge is necessary to detect mistakes in students’ solutions, while pedagogical content knowledge is necessary to uncover typical misconceptions related to the content of a task (Philipp 2018). Using the example displayed in Fig. 1, detecting that two of the six subtractions have been solved incorrectly requires content knowledge about subtraction. Realizing that the student subtracted the smaller from the larger number digit by digit in the two incorrect tasks, and categorizing this mistake as a typical mistake in multi-digit subtraction requires specific pedagogical content knowledge. The example illustrates that with regard to teachers’ diagnostic competencies in primary school mathematics, it is reasonable to expect a larger variation in teachers’ pedagogical content knowledge than in their content knowledge because the mathematical content is fairly basic. In this study, we therefore aim at assisting (prospective) teachers in applying their pedagogical content knowledge by providing a competence model which helps to differentiate different levels of mathematical competence and to map misconceptions to these levels (see 1.1).

In summary, both applying relevant pedagogical content knowledge and performing relevant diagnostic activities (see 1.3) seem important for diagnostic processes and outcomes. One way of investigating the relevance of these facets is to foster both facets during a diagnostic situation and compare their impact on the diagnostic process and outcome. A method to stimulate these knowledge facets during the diagnostic situation is scaffolding.

### Providing Instructional Support Through Scaffolding

Assessing students’ mathematical abilities and misconceptions based on selecting and evaluating tasks is complex, and might overwhelm prospective teachers who do not have sufficient professional experience. Therefore, providing instructional guidance seems necessary, especially during the early phases of competence acquisition (Smetana and Bell 2012). Scaffolding is a process-orientated form of instructional guidance; it means supporting learners while they solve a complex task by reducing and regulating the complexity (Wood et al. 1976). The idea of scaffolding is strongly connected with Vygotsky’s (1978) Zone of Proximal Development. The support is supposed to step in between “the actual development level as determined by independent problem solving and the level of potential development as determined through problem-solving under […] guidance” (Vygotsky 1978, p. 83). With the help of scaffolding, learners are guided to solve tasks that they would not be able to solve on their own (van de Pol et al. 2010).

In a meta-analysis, Chernikova et al. (2020a) found that scaffolding is an effective way to foster diagnostic competencies in teacher education, but the authors also note that there are still few studies comparing different *kinds* of scaffolding. For fostering prospective primary school teachers’ competencies to assess students’ mathematical abilities and misconceptions based on their written task solutions, two kinds of scaffolding seem to be promising: the first supports the application of relevant pedagogical content knowledge (see 1.4), the second supports diagnostic activities (see 1.3).

The first kind of scaffolding (hereafter: *content-related scaffolding*) can include, for example, stimulating knowledge about characteristic abilities and mistakes at different levels of students’ competence. Such knowledge is particularly relevant when the diagnostic situation does not require the comparison of students’ overall competencies in relation to one another (rank-order assessment) but to assess individual students’ level of mathematical competencies relative to a normative competence model and to classify individual students’ specific mathematical abilities and misconceptions. Experimental studies showed that directly instructing prospective teachers in specific pedagogical content knowledge improved their accuracy in assessing task difficulties (Ostermann et al. 2018) or task features (Rieu et al. 2022; Schreiter et al. 2022). Consequently, interventions that provide relevant knowledge have the potential to foster prospective teachers’ diagnostic competencies effectively even in short interventions during university courses. More domain-general research on instructional guidance also suggests that content-related scaffolding can support learning effectively (Bulu and Pedersen 2010; de Jong and van Joolingen 1998; Rieber et al. 2004; Sandoval 2003; Zembal-Saul et al. 2002). Studies using this kind of scaffolding showed that presenting the support continuously during the learning situation helps learners to apply relevant knowledge to solve the task and to improve their competencies in reflecting their judgments.

The second kind of scaffolding (hereafter: *strategic scaffolding*) that might foster diagnostic competencies supports relevant diagnostic activities. Such scaffolding could, for example, encourage learners to generate hypotheses or draw conclusions (de Jong and van Joolingen 1998). Studies that focused on teachers’ diagnostic activities found that prospective teachers tend to collect and describe a lot of information, but rarely integrate the information to state hypotheses or to draw conclusions (Codreanu et al. 2021; Stürmer et al. 2013; Wildgans-Lang et al. 2020). These studies suggest that scaffolding should specifically stimulate these diagnostic activities.

In conclusion, there are good theoretical and some empirical reasons to assume that both content-related scaffolding and strategic scaffolding can support specific facets of prospective teachers’ diagnostic processes (e.g., Codreanu et al. 2021; Fischer et al. 2014; Heinrichs 2015; Ostermann et al. 2018; Wildgans-Lang et al. 2020). Although empirical studies have not directly compared the effectiveness of the two types of scaffolding, we assume that content-related scaffolding is more effective than strategic scaffolding, especially when the diagnostic situation requires assessing content-specific facets (e.g., misconceptions in mathematics).

### The Present Study

The general goal of our research is to develop a simulation for assessing and fostering diagnostic competencies of prospective primary school teachers of mathematics. An evaluation of an initial version of our simulation showed that preservice teachers rated the simulation as suitable with regard to authenticity and presence (Wildgans-Lang et al. 2020). While the initial version of the simulation did not yet contain specific instructional support, we developed two new versions of the simulation with scaffolding.

The present study had three aims: First, we wanted to evaluate the two newly developed versions of the simulation (with scaffolding) regarding perceived authenticity, presence, and cognitive load. To that end, we compared participants’ perceptions of the simulation between groups that worked with different versions of the simulation. We addressed the following research question:

RQ1: Does the implementation of different kinds of scaffolding in the simulation affect individuals’ perception of presence, authenticity, and cognitive load, relative to the simulation without scaffolding?

The second aim of this study was to investigate whether using the simulation had a positive effect on participants’ diagnostic accuracy. We addressed the following question:

RQ2: Does using the simulation increase prospective primary school teachers’ accuracy regarding students’ competence levels and their specific misconceptions?

We used a pre-post-test design to compare the accuracy of a group of participants that received an intervention with the simulation to data from a control group that did not receive any intervention.

Finally, the third aim of this study was to investigate the specific effects of two different kinds of scaffolding that we had implemented in the simulation, namely content-related scaffolding and strategic scaffolding (see 1.4). The content-related scaffolding supported knowledge about the hierarchy of primary school students’ competencies in mathematics (as described in a competence model), whereas strategic scaffolding aimed at supporting the diagnostic process by prompting relevant diagnostic activities. Two research questions addressed the effects of these two kinds of scaffolding:

RQ3: a) Does scaffolding (either content-related or strategic) have a positive effect on diagnostic accuracy regarding students’ competence levels and their specific misconceptions? b) Is content-related scaffolding more effective than strategic scaffolding?

Our hypothesis was that scaffolding positively influences participants’ diagnostic process while they work with the simulation, which is reflected in a higher accuracy compared to using the simulation without scaffolding. Moreover, we expected content-related scaffolding to be more effective than strategic scaffolding.

## Methods

### The Simulation

The simulation was implemented by using the CASUS e‑learning software by the non-profit company INSTRUCT gGmbH as a platform. The software provides a framework for building authentic diagnostic cases and has been created in cooperation with researchers in medical education and learning science. An initial version of the simulation is described in Wildgans-Lang et al. (2022) and was used in the study of Wildgans-Lang et al. (2020). In the simulation, the learners assess virtual students’ mathematical competencies by selecting tasks and viewing the virtual students’ written task solutions. The assessment of virtual students’ competencies in the simulation includes assigning them to a competence level and detecting a possible mathematical misconception. To obtain information about a virtual student’s competencies, participants can select blank tasks from a given portfolio. After deciding on a task, the virtual student’s solution appears. The order of task selection depends on the participants’ individual choices. The participants can stop the process of task selection anytime in order to finish an assessment. After selecting a task, it is possible to take notes while the student’s solution is presented. The notes can be viewed anytime until the final assessment.

The virtual students’ task portfolios contain 23 to 29 different task solutions. An important feature of the simulation is that the tasks and the task solutions were taken from a pilot run of national large-scale assessment among third-graders in Germany (VERA‑3; “Vergleichsarbeiten”). This means that the tasks can be assigned to one of five competence levels according to a competence model, which has been validated from a theoretical and empirical perspective (Reiss and Winkelmann 2009; Reiss et al. 2012). Moreover, the task solutions stem from real students that have participated in the VERA‑3 pilot study, and are therefore authentic. The students’ competence levels can be assigned to one of the five competence levels of the model. Specifically, the empirical scales used in VERA‑3 were designed such that students could be assigned to a certain competence level based on their performances. Students in the VERA-3 study solved more than 50% of the tasks that are below or at the same level as their competence level correctly, and less than 50% of the tasks above this level (Stanat et al. 2012). Similar to this empirical data from VERA‑3, and to reduce complexity, the simulation was constructed such that virtual students who are on a certain competence level would correctly solve most (on average: 74%) of the tasks below or at the same level as their competence level, and would correctly solve only few (on average: 11%) of the tasks above this level. To further reduce the complexity of the simulation, we only implemented tasks that belong to the mathematical content areas “numbers and operations” and “patterns and structures”.

**Fig. 2:**
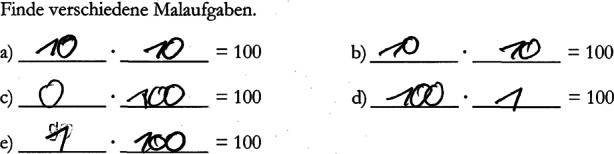
Example of an original VERA‑3 task solution showing a misconception about the use of the number zero

To construct the virtual students’ portfolios, we carefully selected written task solutions from the VERA‑3 item pool such that the solutions revealed specific competencies and misconceptions that were relevant for the assessments. In particular, each virtual student was assigned one major misconception (e.g., systematic mistakes when subtracting with carrying or when calculating with the number zero; see Fig. 1 and 2), based on typical misconceptions as described in the mathematics education literature (Padberg and Benz 2011).

In the simulation, the tasks that can be selected are grouped by the two content areas (numbers and operations or patterns and structures) and by their broad difficulty (“rather easy” or “rather difficult”). The “rather easy” category included tasks at the first two competence levels, and the “rather difficult” category included tasks at competence levels three to five. Only the broad categories but not the specific competence levels of tasks were visible for participants.

### Sample

This study is based on data from a total of 258 prospective primary school teachers (227 female, 30 male, 1 did not specify) who were students at two German universities. Their average age was 22.72 (*SD* = 3.43) and their median university semester was the fourth, ranging from the second to the eighth semester (*IQR* = 4–3). From the original sample of 334 participants, 76 dropped out during the study. Presumably, this large dropout was mostly due to the fact that all assessments had to be conducted online, due to the global Covid-19-pandemic.

Data were collected separately in two waves. First, we collected data from 62 participants who formed the *intervention group*. These participants received the intervention with the simulation. The participants of this group either took part in the study as part of online courses within their curriculum or voluntarily for compensation of € 10 per hour. These participants were randomly assigned to three conditions. One subgroup worked with the simulation that included content-related scaffolding (*content intervention group, n* *=* 24), another subgroup worked with the simulation with strategic scaffolding (*strategy intervention group, n* *=* 18). The third subgroup worked with the simulation without scaffolding (*no-scaffold intervention group, n* *=* 20). Before excluding dropouts, each group consisted of 30 participants. Although in absolute terms, the number of dropouts in the strategy intervention group (12) was larger than the number of dropouts in the content intervention group (6), this difference was not significant (*X*^*2*^(1) = 2.00, *p* = 0.16), so that we assume that the dropouts were not systematic.

Second, we collected data from a larger group of 196 students who participated in pre- and post-testing but who did not receive any intervention. The participants of this group took part in the study as part of an online course within their curriculum. We recruited a larger sample in order to conduct further process data analyses (not reported here). In the current study, this group serves as a general *control group* that allows evaluating the effect of the simulation regardless of scaffolding. Before excluding dropouts, this group consisted of 244 participants.

The non-randomized allocation between control and intervention groups and the small sizes of the intervention groups limit the conclusions that can be drawn from significance tests of group differences. Therefore, and considering current debates in the literature, we based some interpretations not solely on significance thresholds but also discuss the descriptive data (Bakker et al. 2019), while being aware of the general limitations in generalizing our results.

### Instruments

#### Presence

The participants’ perceived presence was assessed with an adapted scale based on Frank (2015), Seidel et al. (2011), and Vorderer et al. (2004). The scale was introduced by the stimulus “Please assess the following statements” and was measured on a 5-point Likert scale (i.e. 1 = “I totally disagree” to 5 = “I totally agree”). The scale consisted of three items, for example, “While assessing in the simulation I concentrated fully on the situation”. The reliability of this scale was high (Cronbach’s *α* = 0.81).

#### Authenticity

Authenticity was measured with an adapted scale based on Seidel et al. (2010) and Schubert et al. (2001). Like Presence, the scale was introduced by the stimulus “Please assess the following statements” and was measured on the same 5‑point Likert scale. The scale consisted of three items, such as “Working in the simulation seemed like a real professional challenge”. Reliability was also high (Cronbach’s *α* = 0.79).

#### Perceived Cognitive Load

Regarding the participants’ perceived cognitive load, we used a scale by Eysink et al. (2009) which measures extreneous cognitive load in digital learning environments. Participants were asked to rate their perceived cognitive load on a 5-point-Likert-scale (i.e., 1 = “very easy” to 5 = “very difficult”). The scale consisted of three items, such as “How easy or difficult is it for you to distinguish between important and unimportant information in the learning environment?”. The reliability of this scale was sufficiently high (Cronbach’s *α* = 0.71).

#### Diagnostic Accuracy

We assessed two facets of accuracy: 1) accuracy in determining a virtual student’s mathematical competence level, and 2) accuracy in determining a virtual student’s misconception. Participants were asked to choose the correct competence level for each virtual student in a single-choice item (with the five competence levels as possible answers, without any content-related description of the levels). The item was introduced by the stimulus “Please select the correct competence level”. Participants were also asked to choose each virtual student’s misconception in a single-choice item (with 13 possible answers). This item was introduced by the stimulus “Please select the statement that you think most likely fits the student” and an exemplary answer was “The student has difficulties regarding the place-value system”. The answers for each facet of accuracy (competence level and misconception) were coded as 1 if the choice was correct, and 0 otherwise.

#### Pretest and Posttest

The pretest and posttest each consisted of assessing one specific virtual student within the simulation. The virtual student in the pretest was at competence level three and the virtual student in the posttest was at level four. Accuracy for the pre- and posttest was assessed. During pretest and posttest, none of the groups received any scaffolding.

### Procedure

Before the testing, all participants were introduced to the competence model of Reiss and Winkelmann (2009), which shows the hierarchy of the different competence levels (without specific descriptions of content areas such as “numbers and operations”; for details, see Reiss and Winkelmann, 2009). The participants were also informed about the aim of their assessments, that is, to assign virtual students to a competence level and to identify their mathematical misconceptions. Furthermore, participants were instructed to proceed as long with a virtual student until they were sure about their assessment and to take notes about their thoughts while they were assessing.

After the introduction, participants completed a pretest (30 min) and then received the intervention with the simulation (60 min; for the three intervention groups only). After that, they completed the posttest (30 min). Finally, participants filled in a questionnaire that included questions about their perceived presence, authenticity, and perceived cognitive load (5 min). The control group only took part in pre- and post-testing (with a time interval of 60 min in between) but did not receive any intervention. All assessments were conducted online due to the global Covid-19-pandemic.

#### Interventions

The three intervention groups received a 60-minute intervention between the pretest and the posttest. The intervention consisted of the assessment of up to seven virtual students in the simulation, who varied in their competence levels and misconceptions. Virtual students were presented in an ordered list, and participants were asked to assess these virtual students in the given order. The three intervention groups used different versions of the simulation. The content intervention group used a version that contained content-related scaffolding (see 2.4.3), the strategy intervention group used a version that contained strategic scaffolding (see 2.4.3), and the no-scaffold intervention group used a version without any additional support. In all versions, the correct assessment was shown after a participant had completed the assessment of a virtual student.

#### Scaffolding

The content-related scaffolding contained detailed information about the underlying competence model. When participants in the content intervention group decided to select a task from one of the two available areas “patterns and structures” or “numbers and operations”, descriptions of the competence levels for the selected area were shown during the task selection. The description remained visible until the participant completed evaluating the solution of the selected task. Table 1 shows the content that was presented after participants decided to select a task from the area “patterns and structures”.

**Table 1:** The content-related scaffold in the area “patterns and structures” contained the following information about the competence levels

Competence level	
1	*Understanding easy patterns (e.g., doubling); Understanding and continuing very easy geometric patterns*
2	*Continuing easy number sequences; Detecting incorrect entries in number sequences; Understanding the structure of patterns in easy graphical or numerical sequences*
3	*Understanding of structures in more complex patterns; Continuing more complex patterns; Understanding and interpreting proportional mappings*
4	*Analyzing and continuing complex patterns; Understanding connections between different representations (e.g., graphical, numerical); Using proportional mappings to model and solve word problems*
5	*Proficient in dealing with complex number sequences; Understanding patterns even when different operations are combined; Constructing arithmetic patterns based on given criteria; Developing their own solving strategies*

The strategic scaffolding contained information about important diagnostic activities (see 1.3). The support that was presented to the strategy intervention group is shown in Table 2. The strategic scaffolding was implemented at the same location on the screen as the content-related scaffolding.

**Table 2:** The strategic scaffold contained the following information

1. Problem identification and questioning:* If you detect a mistake in the student’s solution, think about possible reasons that could have caused this error*
2. Hypothesis generation:* State hypotheses about possible misconceptions and competence levels*
3. Choosing tasks and evaluating solutions:* Try to choose tasks based on your hypotheses that can support or falsify your hypotheses*
4. Drawing conclusions:* Based on your evaluations, make a decision about the student’s competence level and misconception*

Both kinds of scaffolding were introduced by the stimulus: “The following hints could be helpful for the assessment.”

### Data and Statistical Analysis

All data transformations and statistical analyses were conducted in *R* (R Core Team, 2008).

For the first research question (potential differences in participants’ perceptions of the different versions of the simulation), we compared the scores for presence, authenticity and perceived cognitive load between the intervention groups that worked with the simulation by using ANOVAs with group (no-scaffold intervention group/strategy intervention group/content intervention group) as a factor.

For the second and third research questions (effects of the intervention and of different kinds of scaffolding on accuracy), we conducted two logistic regression analyses (one analysis for accuracy in determining a virtual student’s mathematical competence level, and another analysis for accuracy in determining a virtual student’s misconception). In each analysis, accuracy in the posttest was the dependent variable and contrasts between specific groups were the independent variables (see Table 3 for the underlying contrast matrix). We included accuracy in the pretest as a control variable in each analysis. The reported odds ratios represent the change in the odds for a correct answer in the posttest depending on the contrasts. The first contrast compares the control group to the intervention group (addressing RQ2). The second contrast compares the no-scaffold intervention group to the two scaffold intervention groups (addressing RQ3a), and the third contrast compares the strategy intervention group to the content intervention group (addressing RQ3b).

**Table 3:** Contrast matrix

	Control → Intervention (RQ2)	No-Scaffold → Scaffold (RQ3a)	Strategy → Content (RQ3b)
Control Group	−1	0	0
No-Scaffold Intervention Group	1/3	−1	0
Strategy Intervention Group	1/3	1/2	−1
Content Intervention Group	1/3	1/2	1

## Results

### Participants’ Perception of the Simulation

The scores for presence, authenticity, and perceived cognitive load for each of the intervention groups are shown in Table 4. All groups rated presence and authenticity as relatively high and their perceived cognitive load as relatively low, which means that the simulation was suitable for providing realistic diagnostic situations and that operating with the simulation did not result in cognitive overload.

**Table 4:** Presence, authenticity, and perceived cognitive load of the intervention groups (all scales ranging from 1 to 5)

	Presence		Authenticity		Perceived cognitive load	
	*M*	*SD*	*M*	*SD*	*M*	*SD*
No-Scaffold Intervention Group	4.11	0.70	3.48	0.66	2.73	0.87
Strategy Intervention Group	3.77	0.75	3.90	0.69	2.88	0.83
Content Intervention Group	4.14	0.59	3.70	0.47	2.84	0.58

Differences between the groups were relatively small. The strategy intervention group reported a slightly lower presence than the other groups. However, the differences between the groups were small and not statistically significant (*F*(2,55) = 1.62, *p* *=* 0.21,* η*^*2*^ = 0.06). The no-scaffold intervention group perceived the simulation as slightly less authentic compared to the other groups. Again, the group differences were not significant (*F*(2,56) = 2.11, *p* *=* 0.13, *η*^*2*^ = 0.07). In terms of perceived cognitive load, the scores differed very little between the groups, and these differences were, again, small and not significant (*F*(2,56) = 0.18, *p* *=* 0.84, *η*^*2*^ = 0.01).

Together, these results suggest that the implementation of scaffolding did not make the simulation significantly less authentic or reduced participants’ presence. Participants’ perceived cognitive load did also not substantially differ between the three intervention groups.

### Effects of the Intervention

The descriptive results for all groups are displayed in Table 5. The solution rates suggest that the difficulty level of pretest and posttest was appropriate. Because the pretest solution rates differed between the groups, we included the pretest value as a control variable in further analyses. The odds ratios reported in Table 6 inform about the effects of the pretest accuracy and between the contrasted groups—an odds ratio below one indicates a negative effect of the predictor and an odds ratio above one indicates a positive effect.

**Table 5:** Solution rates for the diagnostic accuracy items in pretest and posttest per group

	Competence level		Misconception	
	Pretest	Posttest	Pretest	Posttest
Control Group	0.53	0.51	0.47	0.52
No-Scaffold Intervention Group	0.65	0.60	0.45	0.40
Strategy Intervention Group	0.56	0.67	0.33	0.33
Content Intervention Group	0.38	0.71	0.33	0.63

**Table 6:** Parameter estimates for the logistic regression analysis predicting the odds of choosing the correct competence level in the posttest

Predictor	*OR*	*CI*	*p*
Pretest Accuracy	1.31	0.79–2.16	0.294
Control → Intervention^a^	1.62	1.04–2.57	0.036
No-Scaffold → Scaffold	1.34	0.63–2.82	0.444
Strategy → Content	1.13	0.58–2.20	0.719

^a^This is the contrast comparing the three intervention groups to the control group as shown in Table 3 (analogously for the other predictors)

We expected that the three intervention groups together would make better assessments than the control group without intervention. Indeed, this was true with regard to the accuracy in terms of the competence level. The contrast analysis with pretest as a control variable and posttest as the dependent variable resulted in 62% higher odds for the intervention group participants of choosing the correct competence level in the posttest compared to the control group, *p* = 0.036 (see Table 6, first contrast).

Regarding accuracy in assessing students’ misconceptions, the intervention group was 19% less likely to answer the posttest item correctly compared to the control group while controlling for pretest accuracy (see Table 7, first contrast). This was unexpected. However, the difference was not statistically significant, *p* = 0.367.

**Table 7:** Parameter estimates for the logistic regression analysis predicting the odds of choosing the correct misconception in the posttest

Predictor	*OR*	*CI*	*p*
Pretest accuracy	1.30	0.79–2.15	0.304
Control → Intervention^a^	0.81	0.52–1.27	0.367
No-Scaffold → Scaffold	1.26	0.61–2.68	0.539
Strategy → Content	1.83	0.98–3.58	0.065

^a^This is the contrast comparing the three intervention groups to the control group as shown in Table 3 (analogously for the other predictors)

### Effects of Scaffolding

The third research question was whether scaffolding can enhance the learning effect with the simulation. We expected that scaffolding, in general, would have a positive impact on the learning outcome. Moreover, we expected that content-related scaffolding would be more effective than strategic scaffolding. The results in Table 6 and 7 support our hypotheses only on the descriptive level. Participants in the intervention groups with scaffolding (together) were 34% more likely to choose the appropriate competence level in the posttest than participants in the no-scaffold intervention group (after controlling for the pretest; see Table 6, second contrast), but the difference was not significant, *p* = 0.444. In terms of the misconception, they were 26% more likely to answer the posttest item correctly compared to the no-scaffold intervention group (see Table 7, second contrast). However, this difference was again not significant, *p* = 0.539. These results mean that participants in both scaffold intervention groups (strategy intervention group and content intervention group) showed a tendency of increased assessment accuracy compared to participants in the no-scaffold intervention group, but all differences were not statistically significant.

When contrasting the strategy intervention group and the content intervention group, the descriptive data suggest that a participant in the content intervention group was 13% more likely to choose the correct competence level, *p* = 0.719, and 83% more likely to choose the appropriate misconception in the posttest compared to a participant in the strategy intervention group,* p* = 0.065 (see Tables 6 and 7, third contrast). However, these differences did, again, not reach statistical significance. This result suggests that the intervention with content-related scaffolding tended to have a stronger effect on the accuracy when detecting students’ misconceptions compared to the intervention with strategic scaffolding.

## Discussion

Our research aims at developing a simulation to foster prospective mathematics teachers’ diagnostic competencies. In the simulation, participants can assess virtual students’ mathematical competencies based on written task solutions. In this study, we evaluated different versions of our simulation with different types of scaffolding to identify promising types of instructional support. We evaluated the effects of these simulations on participants’ perception of the simulation and their diagnostic accuracy.

### Participants’ Perception of the Simulation

Three different versions of the simulation were first evaluated in terms of participants’ overall perception. Participants’ ratings in terms of their perceived presence, authenticity and cognitive load appeared to be in the desired range, and did not differ greatly between the different versions of the simulation. This result suggests that these versions can be regarded as comparably well suited for simulating real diagnostic situations.

It should be noted that the items for measuring authenticity relate primarily to the similarity of the simulation to a real professional challenge. Since the participants in this study did not yet have much professional experience, the absolute ratings of authenticity may reflect their subjectively perceived authenticity, rather than the objective authenticity with which our digital tool simulated a professional situation. It would be interesting to find out if experienced teachers would rate authenticity in a similar range. In any case, the material we used to design the simulation can be regarded as authentic since the task solutions stem from real students (see Method section).

### Effects of the Interventions

Our second research question was whether interventions with the simulation can enhance participants’ diagnostic processes and outcomes. Participants who received a 60-minute intervention with the simulation (with or without scaffolding) improved their performance in matching virtual students’ competencies to the appropriate competence level compared to a control group that did not receive any intervention. This result suggests using the simulation can be effective with respect to the assessment of students’ overall mathematical competencies.

Yet, the descriptive results indicated that only the groups that received scaffolding during the intervention, especially the group with content-related scaffolding, contributed to this effect, and that using the simulation without scaffolding did not improve participants’ diagnostic accuracy. Although the latter finding was not as expected, it is actually in line with findings from a meta-study comparing the effects of instructional support on learning outcomes during discovery learning (Alfieri et al. 2011)—and simulation-based learning can be seen as a special case of discovery learning. One finding of Alfieri et al. (2011) was that discovery learning without instructional support was less effective than direct instruction whereas discovery learning with instructional support was more effective than direct instruction. However, in view of evidence that simulation-based learning is highly effective (Chernikova et al. 2020b), we had expected notable learning effects from the simulation even in the version without scaffolding. Apparently, participants were able to benefit from our simulation only when provided with additional instructional support, particularly on the content level. Several reasons may have contributed to this result. Perhaps, participants had too little pedagogical content knowledge regarding the specific content area (numbers and operations, patterns and structures) so that they were not able to activate their knowledge in the simulation. Moreover, the intervention time was maybe too short (60 min) to expect large learning gains. We do not consider the lack of a learning effect of the simulation without scaffolding in our study as a strong indicator against the use of simulations in general, in view of current evidence supporting the effectiveness of simulations (Chernikova et al. 2020b). Nevertheless, we need to critically rethink the support elements that need to be implemented in the simulation to actually make it effective for learning.

### Effects of Scaffolding

The simulations with scaffolding tended to have stronger effects on diagnostic accuracy than the simulation without scaffolding. The results also hint to differences in the effects between the two kinds of scaffolding we used in this study. Previous research has already established that the effects of scaffolding can depend on several characteristics of the sample and the particular kind of scaffolding (Belland et al. 2017; Chernikova et al. 2020a; Hmelo-Silver et al. 2007). For example, unexperienced learners, such as prospective teachers, may profit more from scaffolding that provides higher levels of guidance, whereas more experienced learners can benefit more from types of scaffolding that require a higher level of self-regulation.

Our results suggest that content-related scaffolding has more potential to support participants’ diagnostic accuracy than strategic scaffolding, especially regarding the detection of misconceptions. This finding is in line with previous research, as our content-related scaffolding is closely related to the tasks in the simulation and therefore a stronger form of support than strategic scaffolding. Pre-service teachers may have too little previous experience in making assessments to benefit from strategic scaffolding without direct relation to the content of the simulation. In a qualitative analysis of teachers’ diagnostic competencies, Philipp and Leuders (2014) found that teachers mostly refer to their pedagogical content knowledge, such as knowledge about typical misconceptions, during the process of assessing task difficulties and evaluating solutions. The results of our study are in line with this finding: Providing participants with descriptions of students’ competence levels may have helped them to apply their knowledge about students’ mathematical competencies and misconceptions during the diagnostic process in the simulation.

Although we did not find large effects for strategic scaffolding on participants’ competency to assess competence levels or misconceptions, we do not know if strategic scaffolding did have an effect on the occurrence of relevant diagnostic activities. Further analysis of these activities could contribute to our better understanding of factors influencing diagnostic processes. There is initial evidence that the pure *quantity* of specific diagnostic activities is not significantly correlated with accuracy (Codreanu et al. 2021; Wildgans-Lang et al. 2020), but that the *quality* of diagnostic activities is more relevant. Reliably assessing this quality is, however, more challenging from a research perspective.

### Limitations and Future Directions

In this initial approach of implementing scaffolding in our simulation, we assessed the effects of content-related and strategic scaffolding separately, the first coming from a mathematics educational perspective and the second one from an educational psychological perspective. To make the support most effective, a combination of both types of scaffolding could be promising because the two kinds of scaffolding could complement each other. On the other hand, implementing both kinds of scaffolding simultaneously could also cause cognitive overload because of too much textual information.

In further development of the simulation, we aim at adapting the scaffolding to individual learners’ needs. One of the open questions is on which learner characteristics the adaptation should be based (e.g., prior knowledge, motivational variables). To address this question, it would be interesting to compare the use of the simulation and its effectiveness between a larger sample of pre-service teachers and a sample of in-service teachers. Such a study would also allow analyzing how prior knowledge is related to specific diagnostic processes during the simulations.

On a more general note, one benefit of using digital simulations in studying diagnostic processes is the possibility of recording log data. Such data have the potential to describe the diagnostic process in high resolution, although analyzing (complex) log data is another challenge. Learning analytics methods seem promising for that purpose, and first studies show that these methods allow predicting judgement accuracy to a large extent and helps to identify process indicators of promising and problematic sequences of diagnostic activities (Brandl et al. 2021). Analyzing these log data systematically may improve our understanding of the effects of scaffolding and thus ultimately contribute to an evidence-based use of adaptive digital tools in teacher education.
